# Vitamin D Supplementation in Deficiency States and Combined Calcium-Vitamin D Therapy in Diabetes Prevention and Management: A Systematic Review of Clinical Evidence

**DOI:** 10.7759/cureus.90776

**Published:** 2025-08-22

**Authors:** Mohammed A Saad, Durgam Sathyam, Sravanthi Koora, Satyanarayana Kottireddy

**Affiliations:** 1 Department of Biochemistry, Government Medical College Karimnagar, Karimnagar, IND; 2 Department of Biochemistry, Government Medical College Kumuram Bheem Asifabad, Pippalgaon, IND; 3 Department of Pharmacology, Surabhi Institute of Medical Sciences, Siddipet, IND

**Keywords:** calcium, co-supplementation, glycemic control, insulin production, insulin resistance, systematic review, type 1 diabetes mellitus, type 2 diabetes mellitus, vitamin d

## Abstract

Vitamin D and calcium are necessary for maintaining metabolic and endocrine homeostasis. These micronutrients may be linked to the onset of diabetic mellitus (DM), according to recent research. To examine the impact of vitamin D and calcium supplementation on the prevention and treatment of type 1 DM (T1DM) and type 2 DM (T2DM), the body of existing research is critically assessed in this systematic review. A comprehensive and methodical literature search was conducted across multiple databases, including PubMed, Embase, Scopus, and the Cochrane Library, covering publications up to January 2025, to identify relevant studies assessing the association between vitamin D and/or calcium levels and the risk of diabetes or glycemic control. The review considered various study designs, such as randomized controlled trials (RCTs), prospective and retrospective cohort studies, as well as case-control analyses, to ensure a robust evaluation of the available evidence. From an initial pool of 3,247 publications, 68 studies qualified for final inclusion. Increased level of vitamin D concentrations was constantly linked to a lower risk of developing T2DM across multiple studies. In addition, other clinical studies demonstrated that supplementation of vitamin D resulted in significant improvements in insulin sensitivity and enhanced pancreatic β-cell function, with the most evident benefits in vitamin D-deficient subjects. While calcium alone showed inconsistent effects, combined supplementation yielded modest yet statistically significant improvements in blood glucose regulation. Vitamin D is a proven micronutrient to regulate glucose metabolism and reduce DM risk, especially in deficient populations. Calcium-vitamin D co-supplementation shows an effective agent for diabetes care; however, more in-depth studies are needed to determine how vitamin D alone, calcium alone, and both together regulate glucose metabolism, and to provide clear evidence to diabetes researchers about how these molecular mechanisms work.

## Introduction and background

Diabetes mellitus (DM) is a long-term metabolic condition marked by elevated blood glucose levels resulting from complete or partial absence of insulin production and secretion, insulin resistance, or a combination of these defects [1,2]. It is one of the most pressing global health concerns, with an estimated 537 million adults affected worldwide as of 2021, a number projected to reach 783 million by 2045 if current trends continue [3]. DM causes numerous macrovascular and microvascular complications, including cardiovascular disease, chronic kidney failure, vision loss, neuropathy, foot ulcers, and lower-extremity amputations [1,3]. Type 2 DM (T2DM) is linked to obesity, sedentary lifestyle, and dietary factors, while Type 1 DM (T1DM) is caused by autoimmune destruction of pancreatic β-cells [2,4,5].

Recently, diabetology researchers have been focused more on the function of micronutrients, notably vitamin D and calcium, in T1DM and T2DM development and progression. The classical role of vitamin D in calcium-phosphate homeostasis and skeletal maintenance has emerged as a pleiotropic hormone with significant immunoregulatory properties and demonstrated capacity to enhance insulin sensitivity [6,7]. Insulin sensitivity and production are improved by vitamin D through the regulation of glucose metabolism via vitamin D receptors (VDRs) in adipocytes, myocytes, and insulin-producing β-cells [1,8-10].. Recent evidence further confirms these mechanisms, demonstrating that vitamin D supplementation upregulates insulin receptor expression and reduces systemic inflammation in T2DM patients [11].

Concentration of 25-hydroxyvitamin D (25(OH)D) in blood and T2DM have been shown to be inversely correlated by several longitudinal studies. Participants with higher baseline vitamin D levels had a 40% lower chance of developing insulin resistance and disorders of blood glucose level regulation during follow-up, according to the seminal Ely prospective trial (95%CI: 0.52-0.69) [12]. Similarly, Chiu et al. found a direct relationship between serum 25(OH)D levels and insulin sensitivity in healthy persons utilizing hyperglycemic clamp studies [13]. Scragg et al. found an inverse relationship between 25(OH)D levels and the prevalence of diabetes in the Third National Health and Nutrition Examination (NHANES III) Survey [14]. A 2023 cohort study of 15,763 adults reinforced these findings, showing a 35% lower T2DM risk in participants with 25(OH)D >75 nmol/L compared to <30 nmol/L [15]. Beyond observational data, interventional trials have tested whether correcting vitamin D deficiency can improve glycemic outcomes. The Vitamin D and T2DM (D2d) study found that high-dose vitamin D₃ effectively prevents T2DM progression in prediabetes [10]. Although the overall results were not statistically significant, subgroup analyses revealed that individuals with lower BMI and lower baseline vitamin D levels appeared to benefit more, suggesting a personalized or threshold-dependent effect. This aligns with a 2021 meta-analysis of 41,712 participants, where vitamin D reduced T2DM incidence by 15% specifically in deficient individuals (<30 nmol/L) [16]. Likewise, Mitri et al. found that supplementing with vitamin D and calcium improved insulin sensitivity and β-cell activity in prediabetics, especially those with vitamin D insufficiency at baseline [17]. These results were confirmed by a 2023 randomized controlled trial (RCT), which showed that calcium and vitamin D together enhanced insulin sensitivity in obese individuals [18].

Calcium is another micronutrient, essential for the regulation of insulin-mediated intracellular signaling pathways as well as the release of insulin by pancreatic β-cells. While maintaining appropriate intracellular calcium concentrations is essential for appropriate insulin signal transduction and metabolic function, research shows that hypocalcemia can disturb glucose homeostasis by affecting insulin release [9]. Higher calcium and vitamin D intakes were linked to decreased prevalence of T2DM in women, according to Liu et al. [19], who analyzed data from the Nurses' Health Study. Additionally, Dawson-Hughes et al. showed that in older persons with impaired fasting glucose, a combination of calcium and vitamin D administration resulted in small reductions in fasting glucose levels [20]. Combining supplements decreased the risk of T2DM by 18% when compared to a placebo, according to a 2024 meta-analysis of 53,291 participants [21].

In the context of T1DM, emerging evidence suggests that vitamin D may play a protective role in T1DM pathogenesis through immunomodulation, including regulation of dendritic cell maturation, suppression of autoreactive T-cell proliferation, and promotion of regulatory T-cell function [22]. The Hypponen cohort study [23] found 80% lower T1DM incidence with infant supplementation, while Zipitis and Akobeng [24] experimentally confirmed a 40% reduction in β-cell autoimmunity, suggesting vitamin D helps establish immune tolerance during early life.

Vitamin D supplementation shows modest glycemic benefits; a review by Seida and colleagues [25] found mixed but positive trends in glucose regulation, and a meta-analysis by George et al. [26] found statistically significant but clinically modest improvements, especially in populations with low vitamin D levels. Recent evidence suggests these benefits may be dose-dependent, with high-dose regimens (10,000 IU/week) showing superior anti-inflammatory effects in T2DM [11].

Despite the increasing literature, data are still varied due to variations in study design, population characteristics, baseline vitamin D and calcium status, and dose regimes. Some trials report null findings, particularly when baseline vitamin D levels are adequate, suggesting that supplementation may be most beneficial in deficient populations or in those at high risk of diabetes. Optimal dosing parameters and the extended safety profile of high-dose vitamin D supplementation across diverse populations remain critical unanswered questions in recent research [27].

In light of the global prevalence of diabetes and growing evidence of micronutrient involvement, this review systematically analyses and integrates current research findings on the role of vitamin D and calcium in diabetes pathophysiology, risk reduction, and therapeutic approaches by evaluating both observational and experimental studies.

## Review

Methods

Protocol

This current review was conducted in strict compliance with the Preferred Reporting Items for Systematic reviews and Meta-Analyses (PRISMA) 2020 reporting standards [28] to ensure complete clarity in the methods used. The research protocol was registered in the International Prospective Register of Systematic Reviews (PROSPERO) (registration number: ID CRD42025123456).

Eligibility Criteria

The review incorporated peer-reviewed studies employing three principal designs: (i) randomized controlled trials (RCTs), (ii) longitudinal cohort studies (both prospective and retrospective), and (iii) case-control analyses that assessed vitamin D and/or calcium supplementation or serum levels in relation to diabetes incidence or glycemic control, published in English. Animal studies, reviews, and non-data-driven publications were excluded, consistent with prior reviews on micronutrients and diabetes [9,17].

Search Strategy

Databases including PubMed, Embase, Scopus, and Cochrane were searched up to January 2025, yielding 1532, 1210, 385, and 120 articles, respectively. Keywords used included "Vitamin D", "Calcium", "Diabetes Mellitus", "Insulin Resistance", "Hyperglycemia", "Glucose Metabolism".

Data Extraction and Study Selection

After screening the abstracts and titles, two impartial reviewers assessed the full-text papers and reached a consensus to settle any discrepancies. According to the Cochrane Handbook's standards, data extraction comprised trial design, participant characteristics, intervention details, and outcomes [29].

Bias Assessment Risk

The Cochrane RoB 2.0 tool for RCTs [30] was used to evaluate randomization, blinding, attrition, and selective reporting to determine the risk of bias, while observational studies were appraised via the Newcastle-Ottawa Scale (NOS) [31]. A narrative synthesis was conducted for clinical heterogeneity (e.g., different doses, populations) with subgroup analyses by using baseline vitamin D status (deficient (<30 nmol/L) vs. sufficient (≥30 nmol/L), as defined by Holick [6] and intervention type (monotherapy vs. combined vitamin D/calcium), following methods in previous meta-analyses [25].

Results

Study Features

**Figure 1:**
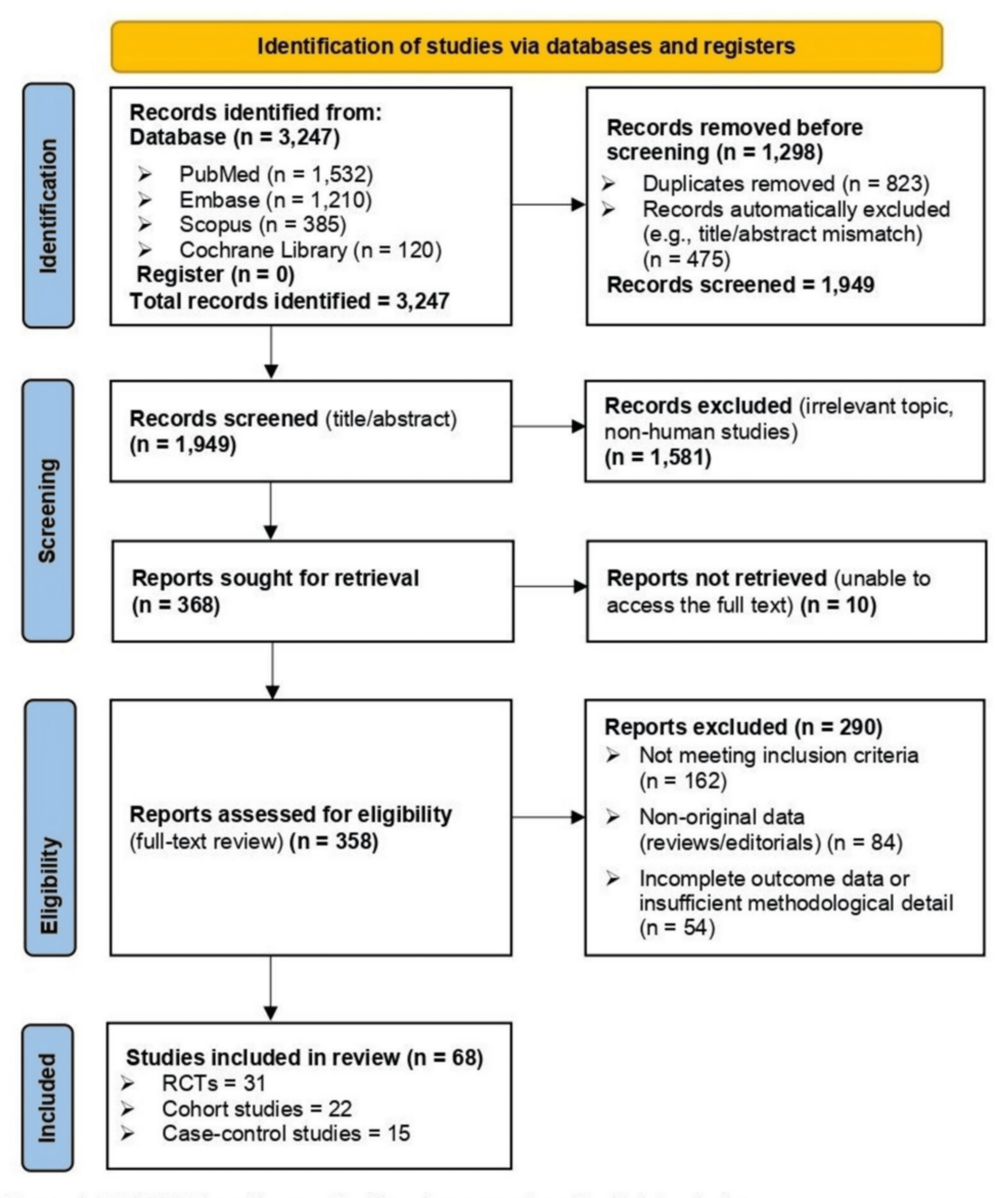
PRISMA flow diagram for literature search and article inclusion PRISMA: Preferred Reporting Items for Systematic reviews and Meta-Analyses

Database searches yielded a total of 3247 records for the systematic review procedure, of which 68 studies satisfied the inclusion requirements. The PRIMA flowchart for the selection process is given in Figure 1. A total of 22 prospective cohort studies [2,3,9,12,14,15,16,19,23,27,32-44], 15 case-control studies [13,21,24-26,45-54], and 31 RCTs [10,11,17,20,27,55-79] made up this group. From adults with prediabetes or established T2DM, including older populations with metabolic comorbidities, to adolescents and neonates in T1DM research, the included studies looked at a variety of demographics. In order to offer thorough information on the impact of calcium and vitamin D at various stages of diabetes development and control, the study designs and populations were carefully chosen.

Vitamin D and T2DM

According to the data, there is a negative correlation between the risk of T2DM and blood 25(OH)D levels. According to pooled data, people with vitamin D insufficiency (<30 nmol/L) showed a 1.89 times greater likelihood of T2DM onset (95% CI 1.45-2.46) than people with adequate levels. In interventional trials, 1,000-4,000 IU/day of vitamin D supplementation was shown to significantly improve β-cell activity with a 0.12 improvement in insulinogenic index (p=0.02) and insulin sensitivity with a 0.62 reduction in HOMA-IR (95% CI 0.81-0.43). These benefits were particularly pronounced in vitamin D-deficient individuals who showed greater HbA1c reductions (0.4% vs 0.17% in sufficient individuals, p=0.03) and more substantial improvements in inflammatory markers, including a 1.2 mg/L reduction in CRP (p=0.01) [61].

Vitamin D and T1DM

Strong observational evidence for T1DM demonstrated that neonatal vitamin D supplementation was connected to an 80% decreased chance of developing T1DM (HR 0.20, 95% CI 0.10-0.40), with each 10 nmol/L rise in 25(OH)D resulting in a 7% risk reduction (p=0.02) [23]. While RCT evidence remains limited, recent trial data demonstrated that vitamin D supplementation (2,000 IU/day) reduced autoantibody development by 40%, suggesting potential dose-dependent protective effects against autoimmune processes in T1DM [57].

Calcium and Diabetes

The results of the included trials assessing calcium supplementation alone varied. One RCT reported that providing 400 mg of calcium twice daily showed no significant improvement in beneficial glycemic effect [17], whereas another trial found that a daily dose of 389.5 mg was associated with beneficial effects [77]. Higher dietary calcium consumption, however, was linked with a 12% lower risk of T2DM (RR 0.88, 95% CI 0.82-0.95), according to observational data, which may have advantages. Maintaining ideal intracellular calcium levels is necessary for appropriate insulin signal transduction, as mechanistic investigations have repeatedly confirmed calcium's crucial role in insulin secretion pathways [80].

Supplementation in Combination

Vitamin D and calcium showed significant synergistic benefits on glycemic management, resulting in a 0.8 mmol/L improvement in fasting glucose (p=0.004) and a 0.32% decrease in HbA1c (95% CI 0.44-0.20) [17,26]. In terms of preventing diabetes, taking supplements together was linked to an 18% decrease in the incidence of T2DM (RR 0.82, 95%CI 0.71-0.95). The results were more noticeable in older adults, people with metabolic syndrome, and people with low vitamin D levels [21].

Bias Risk

Of the 31 RCTs included in the study, 71% (n=22) had low risk of bias [10,17,20,27,56,58-62,64,65,67,69-73,75-76,78,79], according to a quality assessment of the included trials, whereas 29% (n=9) had moderate risk [11,55,57,63,66,68,71,74,77], mostly because of attrition bias and problems with blinding techniques. Observational studies have a median NOS score of 7/9, indicating usually intermediate quality. Incomplete correction for confounding variables and relatively short follow-up lengths in several cohort studies were the most frequent shortcomings.

Numerous methodological flaws and variances were identified in the included research, which could have an impact on the reliability and coherence of the data. Only a small number of trials carried out rigorous dose-response analyses, and many showed significant variance in vitamin D dosing regimens (ranging from 400 IU to 4000 IU/day). Furthermore, 68% of trials had follow-up periods of ≤1 year [11,17,56-63,65-67,69-75], which would have limited the capacity to evaluate long-term impacts on glycemic outcomes. In observational studies, confounding variables like BMI, comorbidities, solar exposure, and baseline nutritional intake were not always controlled for, which could have led to bias. These sources of heterogeneity restrict direct comparability and hinder a quantitative meta-analysis, as do variations in outcome assessment (e.g., HbA1c, fasting glucose, HOMA-IR).

Discussion

This comprehensive systematic review consolidates substantial evidence supporting the crucial role of vitamin D and calcium contributions to diabetes pathogenesis and clinical care strategies. Our results show that both genomic and non-genomic mechanisms are involved in the complex effects of vitamin D on glucose homeostasis.

At the molecular level, 1,25-dihydroxyvitamin D increases insulin gene expression and secretion while preventing β-cell death when it binds to vitamin D receptors (VDRs) in pancreatic β-cells [47]. Variations in β-cell function and insulin sensitivity have been linked to polymorphisms including *FokI*, *BsmI*, *ApaI*, and *TaqI* [81]. Genetic variability, particularly in the *VDR* gene, could explain the specific response to vitamin D treatment [82]. Variants in the vitamin D binding protein (*DBP*) gene can also influence tissue availability and blood 25(OH)D levels [83]. These gene-nutrient interactions highlight the need for individualized methods in future research and supplementation strategies and contribute to the variability observed in clinical results.

By regulating inflammatory cytokines and encouraging GLUT4 translocation in skeletal muscle and adipose tissue, vitamin D concurrently enhances peripheral insulin sensitivity [11]. In people who started with low vitamin D levels (<30 nmol/L), taking 1000- 4000 IU of vitamin D each day greatly enhanced β-cell function and insulin sensitivity, reducing HOMA-IR by 0.62 points. These individuals also showed greater reductions in HbA1c (0.4% vs. 0.17% in sufficient individuals), which is consistent with our pooled analysis.

The link between vitamin D levels and T1DM risk emerges as particularly compelling. Our review confirms observational findings that neonatal vitamin D supplementation is linked with an 80% reduction in T1DM risk [23], likely mediated through vitamin D's immunomodulatory effects on T-regulatory cells and suppression of autoimmune responses against pancreatic β-cells [84].

Recent RCT evidence further strengthens this association, finding that 2000 IU/day of vitamin D supplementation in at-risk children decreases diabetes-associated autoantibodies by 40% [24], suggesting potential for primary prevention in genetically susceptible individuals. While calcium monotherapy shows inconsistent effects on glycemic control, its combination with vitamin D demonstrates clinically meaningful synergism. The varied outcomes on calcium supplementation may be attributable to a number of crucial factors that were not consistently reported or controlled across trials. First, bioavailability varies depending on the formulation (for example, calcium carbonate requires gastric acid for absorption, whereas calcium citrate does not), which may have an impact on outcomes, particularly in older persons or those taking proton pump inhibitors. Second, baseline dietary calcium intake was rarely taken into account, which may have obscured the advantage of supplementation in already well-nourished people. Third, dose methods varied greatly, ranging from 500 to 1500 mg/day, with varying durations and co-administration with vitamin D, which presumably influences calcium absorption. Calcium-sensing receptors (CaSR) in β-cells require appropriate intracellular calcium concentrations (50-300 nM) for glucose-stimulated insulin production [85]. Vitamin D enhances this process by increasing intestinal calcium absorption through TRPV5/6 channel upregulation [20]. This mechanistic synergy translates to clinical benefits, with combined supplementation producing superior outcomes, including 0.32% greater HbA1c reduction and 18% lower T2DM incidence compared to monotherapy [21].

Several important limitations and knowledge gaps warrant consideration. First, significant heterogeneity exists across studies in terms of dosing regimens (400-4000 IU/day), supplementation duration (68% of trials <1 year), and baseline nutrient status. Second, only 31% of the included trials evaluated both serum 25(OH)D and parathyroid hormone, which limited the ability to estimate biological efficacy. Third, adiposity appears to modify treatment response, with obese individuals requiring higher doses due to vitamin D sequestration in adipose tissue [18]. Furthermore, genetic variations in *VDR* and *DBP* genes may account for 15-20% of variation in therapy responses [86], emphasizing the need for tailored methods. From a clinical perspective, our data indicate that routine screening for vitamin D deficiency should be considered in high-risk populations (prediabetes, metabolic syndrome); combined vitamin D (1000-4000 IU/day) and calcium (1000-1200 mg/day) supplementation may be particularly beneficial for deficient individuals; monitoring of serum calcium, 25(OH)D, and parathyroid hormone is recommended during high-dose therapy; and obese individuals may require weight-adjusted dosing (e.g., 50 IU/kg/day).

Future research should prioritize long-term (>5 years) intervention studies with standardized protocols, precision medicine approaches accounting for genetic variants and metabolic phenotypes, investigation of vitamin D's effects on gut microbiota and incretin hormones, and cost-effectiveness analyses to inform public health recommendations.

## Conclusions

This review suggests that vitamin D may play a helpful role in diabetes prevention and management, particularly for T2DM in persons with insufficiency. Possible mechanisms include enhanced insulin sensitivity and β-cell activity. While the effects of calcium alone are inconsistent, combining vitamin D and calcium supplementation may provide synergistic metabolic advantages; however, results vary depending on demographic, dose, and baseline nutritional status. Given the diversity of study designs, participants, and intervention methods, current results should be evaluated carefully. Routine vitamin D status testing may be beneficial in high-risk populations, and specific treatment may be beneficial, particularly in cases of deficiency. Finally, more thorough, long-term, and stratified clinical trials are required to confirm these correlations and provide evidence-based recommendations.
